# An Electro-Oculogram (EOG) Sensor’s Ability to Detect Driver Hypovigilance Using Machine Learning

**DOI:** 10.3390/s23062944

**Published:** 2023-03-08

**Authors:** Suganiya Murugan, Pradeep Kumar Sivakumar, C. Kavitha, Anandhi B, Wen-Cheng Lai

**Affiliations:** 1 Department of Computing Technologies, SRM Institute of Science and Technology—KTR, Chennai 603203, India; suganiya11@gmail.com; 2 Department of Electrical and Electronics Engineering, Vels Institute of Science Technology and Advanced Studies, Chennai 600117, India; pradeepjamcet@gmail.com; 3 Department of Computer Science and Engineering, Sathyabama Institute of Science and Technology, Chennai 600119, India; kavitha4cse@gmail.com; 4 Department of Biomedical Engineering, Agni College of Technology, Chennai 600130, India; anandhiharichandran@gmail.com; 5 Bachelor Program in Industrial Projects, National Yunlin University of Science and Technology, Douliu 640301, Taiwan; 6 Department of Electronic Engineering, National Yunlin University of Science and Technology, Douliu 640301, Taiwan

**Keywords:** drowsiness, visual inattention, machine learning, drowsiness detection, signals

## Abstract

Driving safely is crucial to avoid death, injuries, or financial losses that can be sustained in an accident. Thus, a driver’s physical state should be monitored to prevent accidents, rather than vehicle-based or behavioral measurements, and provide reliable information in this regard. Electrocardiography (ECG), electroencephalography (EEG), electrooculography (EOG), and surface electromyography (sEMG) signals are used to monitor a driver’s physical state during a drive. The purpose of this study was to detect driver hypovigilance (drowsiness, fatigue, as well as visual and cognitive inattention) using signals collected from 10 drivers while they were driving. EOG signals from the driver were preprocessed to remove noise, and 17 features were extracted. ANOVA (analysis of variance) was used to select statistically significant features that were then loaded into a machine learning algorithm. We then reduced the features by using principal component analysis (PCA) and trained three classifiers: support vector machine (SVM), k-nearest neighbor (KNN), and ensemble. A maximum accuracy of 98.7% was obtained for the classification of normal and cognitive classes under the category of two-class detection. Upon considering hypovigilance states as five-class, a maximum accuracy of 90.9% was achieved. In this case, the number of detection classes increased, resulting in a reduction in the accuracy of detecting more driver states. However, with the possibility of incorrect identification and the presence of issues, the ensemble classifier’s performance produced an enhanced accuracy when compared to others.

## 1. Introduction

Drivers at the wheel work for long hours without adequate sleep, leading to poor health and inattentive driving due to chatting on mobile phones, and fatigue are factors that contribute to road accidents. Fatigue is brought on by illness or physical activity, and drowsiness is a prelude to sleep [1]. Inattention is a state of becoming distracted from a current physical activity and occurs in two ways: visual (distracted by sight) and cognitive (distracted by thinking). Road accidents are fatal or result in crippling injuries. According to the road traffic injury and prevention [2] report, nearly 1.25 million people die this way each year, with an average of 3287 deaths a day. A report from the Ministry of Road Transport and Highways indicates that India’s total number of road accidents increased on average by 13.6 percent in 2021 compared to 2020, and the number of fatalities increased by 16.9 percent while the number of injuries increased by 10.4 percent [3]. The driver’s physical state requires careful scrutiny through the continuous monitoring of behavior and body signals in order to bring down the fatality rate and prevent road accidents, ensuring driver safety and well-being [4]. An alternative control method for the lateral motion problem, based on a bounded equivalent function, the vehicle kinematic model, and the Taylor series expansion, has been developed, which ensures both robustness and control accuracy [5]. The author ensured the consistent segmentation of sequential points within behavior actions and provides better accuracy [6]. Kinematics and dynamics models make up the vehicle physics model, which can ensure the precision of short-term prediction [7]. With the likelihood of no system ensuring that manual tabs are kept on the driver, there is a need for the design and incorporation of a hypovigilance detection of components and their incorporation in vehicles.

Hypovigilance is the phenomenon when a driver’s vigilance level lowers over time. Hypovigilance is a state of reduced alertness that impacts concentration while driving [8]. Driver hypovigilance depends on the time of the day controlled by the circadian rhythm, time since sleep, time on task, inadequate sleep, and accumulated lack of sleep [9]. It may be due to either the driver’s drowsiness, inattention, fatigue, or the fact that the driver is hypovigilant. It may be caused by prolonged sleepiness or short-term inattention to impair alertness. It can be detected through behavioral measures that continuously monitor the driver while driving. Physiological signals, which capture electrical activity from the human body, include electroencephalography (EEG), electromyography (EMG), electrooculography (EOG), and electrocardiography (ECG), all of which assist in the detection of driver hypovigilance. Each has its own merits and demerits in terms of real-time use [10]. Compared to behavioral measures, EOG signals are more likely to offer high time resolution, a high recognition rate, and a large variety of features [11]. Researchers have used electrooculography (EOG) in place of video analysis for eye blink detection [12]. Location recommendation models have served the personalized location-based social networks (LBSNs) application referred to as a spatiotemporal individual mobility graph encoding network with group mobility assistance (SIGMA) [13]. A driving situation graph cluster was created by using the formal language of driving cognition based on the software and hardware architecture of an intelligent vehicle by processing information from sensors and driving maps, which was then output to a decision-making module. The decision-making module outputs the output result in the form of cognitive arrow clusters. The detection of eye blinks through a video analysis is fraught with problems due to the driver being constantly on the move, and the eyes are, therefore, invariably in motion. Additionally, the EOG velocity feature detects the closing and opening slopes of the eyes [14]. EEG signals are collected by means of electrodes placed on the scalp and are likely to be noisy and difficult to gather [15]. The uses of blockchain for big data applications in different vertical domains such as smart cities, smart healthcare, smart transportation, and smart grid were reviewed in [16]. In [17], the camera was not only used for the identification of the driver’s face recognition but also to prevent the vehicles from theft.

EOG signals are based on the electrical potential difference between the cornea and the retina during eye movement [18]. Electrooculography (EOG) is a good indicator of driver drowsiness and helps evaluate visual signs of drowsiness [19]. Horizontal and vertical eye movements can be clearly differentiated, as blinks occur only during vertical eyelid movements [20]. Eye movements decrease while the blink rate increases as a person become fatigued [21]. EOG signals comprise two rhythms, namely, slow eye movement (SEM) and rapid eye movement (REM) [22]. REM occurs when one is awake, and SEM when one is drowsy [23]. A longer blink duration and SEM measured using EOG signals are features indicative of drowsiness. Most researchers have combined EEG and EOG for the detection of driver drowsiness [24]. The EEG signal collected from the prefrontal brain region was used in detecting driver’s fatigue with an average accuracy of 0.85 on mixing the accuracy of electrodes FP1 and FP2, which was higher compared to the single electrodes performance [25]. In [26], the final result does not display 100% specificity but rather achieves around 95% accuracy. In electrooculography (EOG), a normal blink is an upward signal change that is followed almost immediately by a downward signal change, which is known as reopening after reaching the peak [27]. EOG features such as blink duration and PERCLOS are ranked higher than any EEG feature [28]. SEM-related EOG features, saccades, blinks, and the energy collected from 22 subjects provide the highest average correlation on a combination of multiple features and vigilance. Detecting a driver’s sleepiness using EEG, EOG, and the contextual information of 30 drivers achieved 79% accuracy for multiclass and 93% for binary classifications [29]. Electrooculography, which chiefly features SEM, detects the driver’s vigilance level while a monotonous task was being carried out [30]. The drawback in recording EOG signals when the driver is at the wheel lies in the difficulty involved in placing electrodes close to the eyes, which is intrusive and hampers movement [31]. A Google glass-based drowsiness detection system was used to monitor eye blink frequency. Drowsiness is detected with rising eye blink frequency, longer braking response times, and increasing lane deviation, all of which serve to alert the driver [32]. The blink detection technique, which was used in videos to detect eye movements has problems with different lighting conditions. The difficulty is detected through the use of an infrared (IR) camera that produces an easily detectable reflection in the eyes [33]. However, the parameters extracted from the video do not correlate with the homologs extracted from electrooculography [34]. The eye blink feature extracted from EOG signals provides the same accuracy as that from a high-frame video. Drowsiness detection using eye blink features extracted from EOG signals, based on the fuzzy method offers an 81.7% correct detection rate and a 13.1% false alarm rate [35]. Researchers have used the EEG cap for the collection of EOG signals [9,13,26]. A forehead EOG device was found to be far more suitable and convenient than a traditional one, from a practical standpoint, for the detection of driving fatigue [26,36]. EOG signals can be measured in a non-invasive and nonintrusive manner [37]. The camera-based driver monitoring system measures eye movements to build a multiple regression model and can predict the driver’s reaction time that it takes for recognition and response to requests in automated driving. Saccades of different sizes and saccadic velocities are correlated with driver performance [38]. In [39], HOS features were proposed for the extraction of features that contain more emotional information when compared to the general statistical features used widely in emotion research using physiological signals. The ease with which EOG signals can be collected, their immunity to slight noises, and the success of EOG-based methods in terms of accuracy have resulted in their use in hypovigilance detection [40].

### 1.1. Motivation

Road accident avoidance before it happens saves lives and lowers the number of injuries, financial losses, and fatalities. This study employed a driving simulator to lower the risk on the road and created an effective detection system utilizing the right approaches and algorithms. The following procedure was involved in creating an intelligent driver hypovigilance detection system. A protocol was initially created for a continuous two-hour driving session with stages of hypovigilance. Three different time periods are evaluated on ten people. To assess the subjective measure, pre- and post-driving surveys were created. EOG electrodes are applied to the individuals’ bodies after a thorough explanation of the protocol.

This physiological measurement can be evaluated using the subject signals. To measure levels of visual and cognitive inattention, weariness, and drowsiness, the subject is permitted to operate a motor vehicle continuously for two hours. When the subject nods off, the session is over. Each session’s signals and video are gathered and pre-processed using the relevant filtering methods. Following the extraction of EOG features, significant features are chosen, features are decreased in dimension, and several classifier techniques are used.

### 1.2. Contribution

The objective of this work was to suggest appropriate methods for recording electrical activity from electro-oculographic (EOG) eye movements to detect driver drowsiness, fatigue, and inattention (visual and cognitive) in order to alert drivers. The novelty of this paper lies in bringing out the data collection for EOG signals from different driver states and the fusion techniques that can be applied in the detection of driver hypovigilance [41]. Very few researchers have used EOG as the physiological measure in driver state detection. This study examines the driver hypovigilance detection using EOG in comparison to the other two-class detection. The study contributes the following findings:

➢ Designing a protocol to induce hypovigilance.
➢ Acquiring the EOG recordings from 10 subjects driving at three different times of the day.
➢ The collected driver physiological information is pre-processed using various filtering techniques.
➢ The classification in five classes (normal, visual inattention, cognitive inattention, fatigue, and drowsy) in which detection performed better with the Ensemble classifier.
➢ The performance of hypovigilance detection by combining the significant features obtained a better accuracy of 90.9%.

The outline of this paper is as follows: Section II details the methodology used in the acquisition of EOG signals. It also provides a description of the system and protocol design, data collection, signal pre-processing, feature extraction, and reduction, and the classification of driver hypovigilance states. Section III presents the results of the ANOVA test and the accuracy obtained, which is based on the performance of driver state classification using several machine learning algorithms. Section IV discusses the results and draws conclusions from the driving experiment for the detection of an EOG-based driver hypovigilance system in real-time.

## 2. Materials and Methods

**Figure 1:**
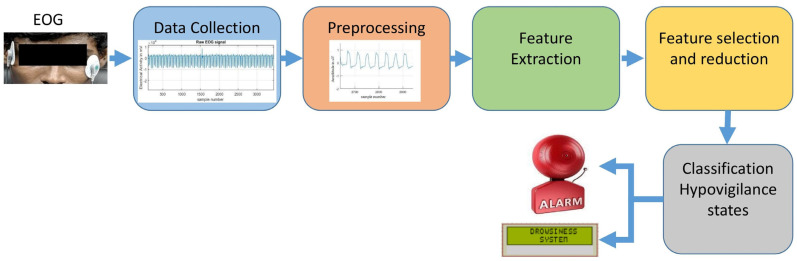
Hypovigilance detection system methodology.

A system that detects driver states (drowsiness, fatigue, visual inattention, and cognitive inattention) using physiological measures has been developed. Figure 1 depicts the methodology of the function involved in the development of a secure system for every vehicle. Initially, a protocol was designed, and the signals collected from the subjects were denoised and features to be extracted. They were then classified using machine learning algorithms based on the driver’s physical state. Finally, the driver was alerted when a behavioral change was detected. 

### 2.1. Experimental Design

**Figure 2:**
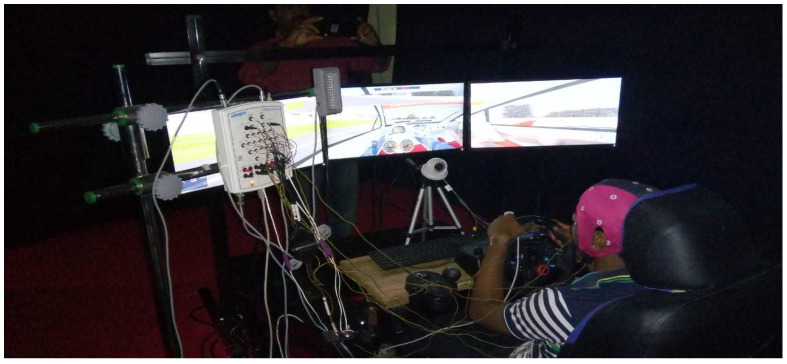
Experimental setup.

The driving task was undertaken at the Artificial Intelligence Lab, VISTAS, Chennai. The lab has a driving simulator with three monitors for game display [42]. The entire room was draped in black to duplicate a nighttime driving environment. A simulator was installed with the Speed Dreams 2.2.1v game for monotonous driving at a speed limit of 70 km/h. A 1-mile low banked oval speedway track was chosen with a constant speed throughout the session, and the driver, feeling fatigued due to the progress made in the session, eventually became downright drowsy. Physiological measures obtained with the use of the Virgo SL-40 PSG device (Allengers, Chennai, India) had the following features: 21-EEG, 2-ECG, 2-EOG, 2-EMG, and 2-SpO2 channel systems; heart rate; abdominal and thoracic body positions; 2 limb movement channels; nasal/oral airflow pressure; and snoring, with 4 auxiliary and 2 bipolar channels. In this study, EOG signals were used for the detection of the hypovigilance states of the subjects. The physiological signals were sampled at 256 Hz. An IR camera in night vision mode captured the driver’s actions for the entire session. Some experiments were seen as having unimodal problems and multi-model problems, which required analysis [43]. The video and signals collected were synced with the time taken. Figure 2 shows the data acquisition system and the experimental testing setup.

**Figure 3:**
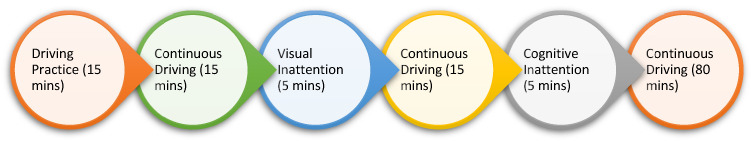
Protocol design.

The driving protocol was designed to test drivers and detect each of the following physical states: normal, visual inattention, cognitive inattention, fatigue, and the gradual stages of drowsiness. The protocol was designed to collect physiological signals over a 2-h continuous driving session (Figure 3).

A total of 30 recordings in all were captured during three different time slots [44] over a 24-h period when the circadian rhythm was low:

➢ 12:00–2:00 a.m.;
➢ 3:00–5:00 a.m.;
➢ 2:00–4:00 p.m.

Each slot consisted of 15 mins of practice driving and 10 mins of normal data collected without driving; 15 mins with driving data collected; 5 mins of data taken for visual inattention (the driver was distracted, via a phone text message requiring a mandatory reply, 3 times in all while driving); 15 mins of continuous driving; followed by 5 mins of data for cognitive inattention (the driver was called upon the phone and asked questions that required thinking through before they were answered and, based on the speed at which the answers were delivered, the level of difficulty in the questions increased); and the final 70 mins of data were used to monitor fatigue and variations in stages of drowsiness (slightly sleepy, moderate sleepy, and extreme sleepy).

### 2.2. Data Collection

**Figure 4:**
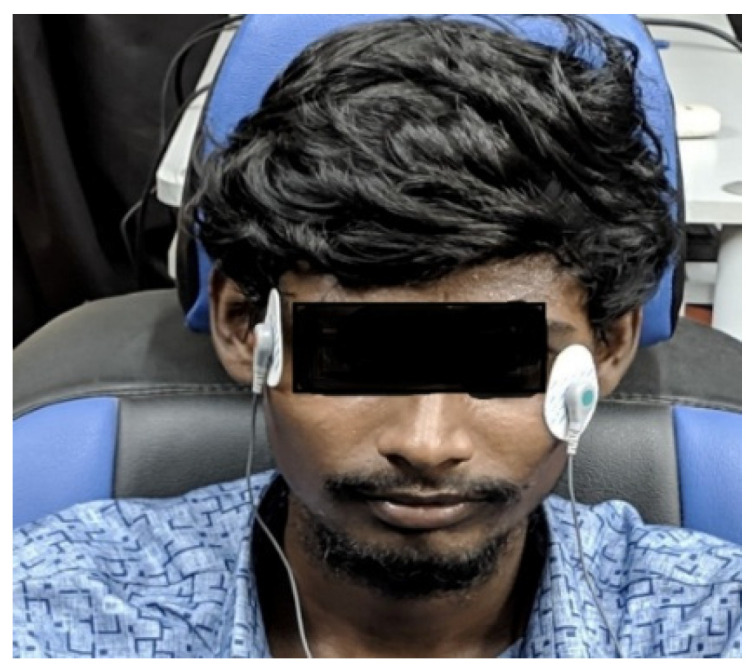
EOG electrode placement.

Data were collected from 10 participants (9 males, 1 female) between 20 and 40 on the basis of the protocol designed, the physiological signals (ECG, EEG, EMG, and EOG), and behavioral (video) data. The protocol was clearly explained to the drivers, who were then asked to fill in a form indicating their willingness to participate in the study. The driver’s personal details and consent form were collected, and an honorarium was paid after the session. The 2-part form handed to the drivers included a pre-questionnaire (on each driver’s sleep criteria) and a post-questionnaire (the experience they had during the 2-h driving session). The forms with the information were collected after the driving session. EOG electrodes were placed near the eyes, to the left and the right (Figure 4). EOG signals were collected while driving and split with respect to the video recorded. The signals were categorized into five classes: normal, drowsy, visual inattention, cognitive inattention, and fatigue.

The placement of EOG electrodes near the left and right eye using conductive gel (Ag/AgCl) and the medical tape was less adhesive on the human skin. They also provided the poor contact of the electrodes during facial movements, leading to the misplacement of non-intrusive electrodes and the occurrence of noises on a larger number in the signal. The wearing of an EEG cap also provided an explanation for the signal contamination, which required proper filtering techniques for the removal of noises without any data loss. This was the real challenge seen in the development of a non-intrusive wearable EOG device for the provision of information in a constant flow with reduced data loss and the development of effective filtering techniques for the removal of artifacts and noise.

### 2.3. Pre-Processing

**Figure 5:**
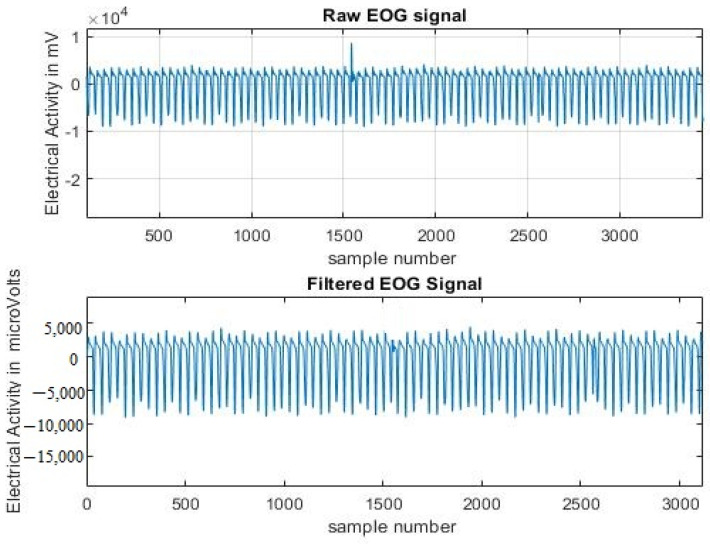
Raw and filtered EOG signal.

The raw EOG signal was contaminated with neuronal sources and artifacts such as facial muscles and body or head movements while speaking, which required proper filtering. The electrodes placed near the eyes are subject to more motion artifacts due to facial expressions, which resulted in baseline wandering and sudden peaks in the signal. In Figure 5, the first plot shows the raw EOG signal with peaks detected and the second plot shows the filtered EOG signal after pre-processing.

Given that the EOG signal information was primarily found in low frequencies, the information was filtered using the Butterworth band pass 6th order filter with a range between 0.1 and 30 Hz and was applied using the sampling frequency at 256 Hz [37]. The cutoff frequency range that was chosen provided useful information for the detection of true internal driver states using EOG. The filtered EOG signal was decomposed using the 8-level Daubechies wavelet (db8). Both EOG signals were taken from the left and right eyes and were pre-processed. Figure 5 displays the raw and filtered EOG signals.

### 2.4. Feature Extraction

In this study, statistical, higher-order statistical (HOS), time-domain, and non-linear features were extracted. Time-frequency domain features, which could not be directly used by themselves, required reductions in terms of dimensions before being directed to a classifier [45]. In all, 16 EOG features comprising 10-time domain features (mean, median, maximum, minimum, root mean square, power, energy, sample entropy, standard deviation, and variance), a higher order of statistical features (skewness, kurtosis), and 5 non-linear features (the Hurst exponent and central tendency measure of nanmean, harmonic mean, mode, and trimmean) were extracted. The EOG features that were extracted, followed by their equations, are shown in Table 1.

## 3. Results

### 3.1. Feature Selection

Features with a significant difference (*p* < 0.05) were selected based on ANOVA. Table 2 shows the EOG features that were selected for classification. They include the mean, maximum, minimum, power, energy, Hurst, variance, sample entropy, nanmean, and mode, all of which were features selected by the ANOVA and fed as inputs into the machine learning algorithm.

### 3.2. Classification

The features were extracted and fed to several classifiers for their best performance. PCA was enabled on the ANOVA-selected features for a reduction in the principal component features for enhanced accuracy. Table 3 depicts EOG’s performance on hypovigilance multi-class detection. It also presents a comparison of the performance obtained by EOG signals collected from the left and right eyes. Features were selected from the ANOVA based on significant differences, and the PCA was applied for feature selection. Additionally, PCA provided support in reducing dimensionality (features) to explore the accuracy of the detection rate. The features chosen for multi-class detection included the mode, Hurst, sample entropy, and mean, which were given to the SVM, KNN, and ensemble classifiers. The EOG for the left eye obtained a maximum accuracy of 94.6% for two classes (normal with visual inattention), 87.9% for three classes (normal, drowsy, and cognitive inattention), 82.7% for four classes (normal, drowsy, visual inattention, cognitive inattention), and 86.6% for five classes (normal, drowsy, visual inattention, cognitive inattention, and fatigue). Similarly, the EOG for the right eye obtained a maximum accuracy of 98.7% for two classes (normal with cognitive inattention), 93.5% for three classes (normal, drowsy, and cognitive inattention), 91.3% for four classes (normal, drowsy, visual inattention, cognitive inattention), and 90.9% for five classes (normal, drowsy, visual, inattention, cognitive inattention and fatigue). Table 3 shows EOG performance in driver hypovigilance detection. Machine learning algorithms (SVM, KNN, and Ensemble) play a vital role in the classification of different classes based on the features trained.

**Figure 6:**
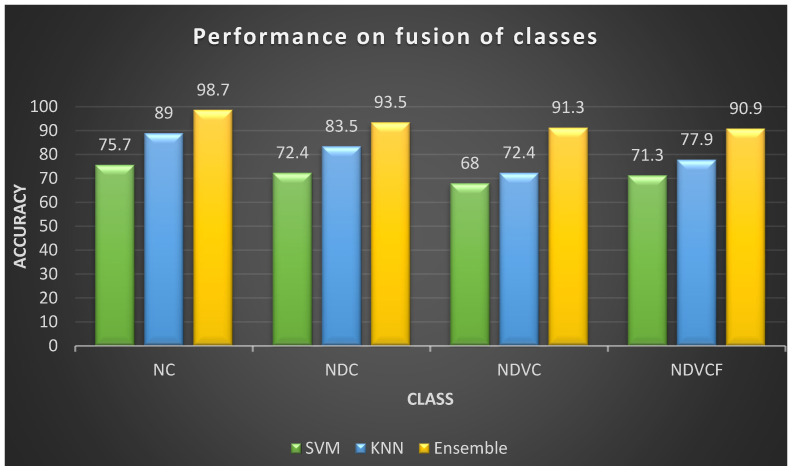
Comparison on classification of EOG in hypovigilance detection.

The inference from the results that the EOG accuracy obtained for multi-class detection was that it was less than that for binary-class detection [46]. Deep learning algorithms enhance performance, just as PCA-reduced features maximize the accuracy of EOG signals. The results showed the mode, Hurst, sample entropy, and mean as the best for all the EOG features. Additionally, the ensemble classifier provided comparatively good performance and accuracy compared to the other classifiers [47], as it used the bagging strategy. This improvement in accuracy came with a much longer response time and fewer error [48]. Figure 6 shows the overall comparison between the classification performances on the fusion of different driver states. Table 2 shows the consolidated maximum accuracy that was obtained for two-class, three-class, four-class, and five-class. The EOG signals found a better detection for visual and cognitive inattention than the other driver states.

**Figure 7:**
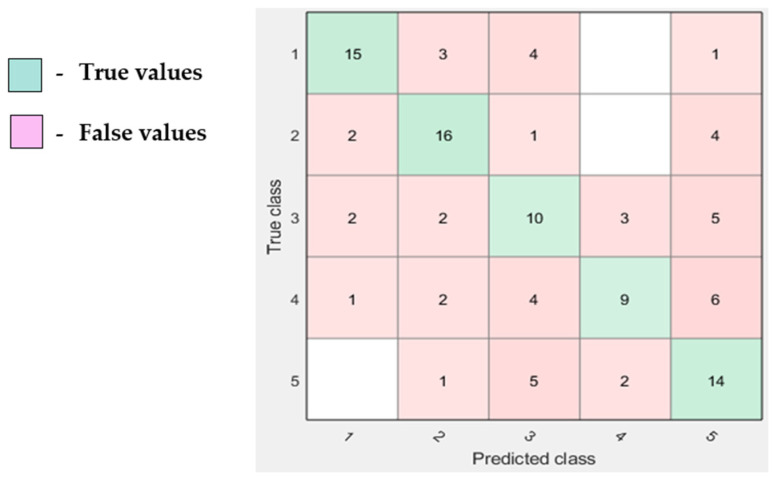
Confusion matrix.

Based on the EOG performance for hypovigilance detection, the maximum accuracy that was obtained was 90.9% from the ensemble classifier. Other corresponding performance metrics such as sensitivity, specificity, precision, and error rate were also calculated using the confusion matrix Figure 7. False positives and false negatives are some of the valuable factors which affect the process of making correct decisions in finding specific problems in the human body [49].

The related performance metrics calculated from the confusion matrix are given by the following:

(i) Accuracy
  ACC = (TP + TN)/(TP + TN + FP + FN)(1)
(ii) Sensitivity (or) Recall
  Recall = TP/(TP + FN)(2)
(iii) Specificity
  Specificity = TN/(TN + FP)(3)
(iv) Precision
  Precision = TP/(TP + FP)(4)
(v) Error Rate
  Error rate = (FP + FN)/(TP + TN + FP + FN)(5)

**Figure 8:**
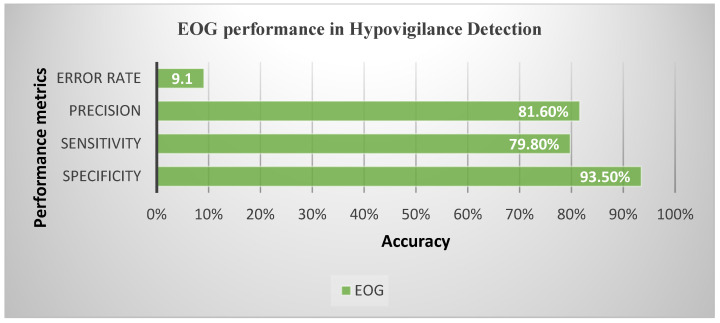
EOG performance metrics for hypovigilance detection.

Figure 8 shows the various performance metrics (sensitivity, specificity, error rate, and precision) were calculated for the maximum accuracy of 90.9%.

Table 4 clearly shows the good performance of EOG in the detection of driver hypovigilance with the application of PCA.

The comparative study from the previous work with the proposed indicated actual performance gain was used. The fusion of five-class detection with the PCA reduced the features and improved the performance of EOG in hypovigilance detection to 90.9%. Compared to the accuracy of two-class detection, hypovigilance had a lower accuracy indicating that the number of detection classes was more. This system used a one-against-all classification approach for multiclass detection, with the advantage that it could detect several driver behavior patterns.

## 4. Discussion

### Performance on Hypovigilance Detection

The focus of this research is on the detection of driver drowsiness, fatigue, and inattention using electrooculography (EOG). This paper, however, combines all three of the states above with five behavior classes for the detection of driver hypovigilance. Chieh et al., 2005 used digital signal differentiation and simple information fusion techniques for the detection of drowsiness, with a detection rate of more than 80% [22]. Similarly, [30] applied fuzzy and supervised learning classification techniques related to the same end producing a good 82% true detection rate and a 13% false alarm rate. This approach obtained a remarkable 86.7% accuracy for driver drowsiness detection (Table 3). For driver fatigue detection, [50] used regression analysis and obtained a correlation coefficient of 0.88 on average. Likewise, using the EOG data of 22 subjects, [9] obtained a high correlation coefficient with an average vigilance reference of up to 0.75 for driver fatigue detection. The indexing technique takes LBP while taking into account data from nearby pixels and is noise-resistant [51,52]. The methods used in this experiment helped obtain an 86.7% accuracy in driver fatigue detection using electrooculography (EOG) compared to earlier experiments (Table 2). Very few researchers have worked on driver cognitive inattention and obtained an overall F1 score of 0.93 [44]. Comparatively speaking, however, the average results obtained from this study for visual and cognitive inattention was 91.1%, indicating an improvement over the results obtained from the earlier experiments on EOG in the detection of different states in which the driver was placed. Table 5 describes the comparison on the performance of proposed model with the existing works.

## 5. Conclusions

This work on driver hypovigilance detection in different states (normal, fatigue, visual inattention, cognitive inattention, and drowsy) acquired from EOG signals will help in the prediction of accidents. The performance on two-class detection was 98.7% which indicated a drastic decrease following an increase in the number of states in which the driver was placed. The maximum accuracy that was obtained to detect driver hypovigilance was 90.9%, with reduced false detection. Electrooculography (EOG) devices can be made less intrusive by turning them into comfortable smart wear items for future driver use. Further research can be taken up with a contribution of electrooculography (EOG) with electroencephalography (EEG) for the detection of different driver states with a reduction in the channels for the achievement of superior performance. A prototype with an alert mechanism could be implemented for the identification of hypovigilance states of the drivers in a real-time driving environment. In the future, experiments need to be performed with a greater number of participants from a wide range of age groups to analyze the results in real-time.

## Figures and Tables

**Table 1:** EOG features and its equations.

Equations for Features	
$\mathrm{Mean}\;\mu_{x\;}=\frac{1}{N}\overset{N}{\underset{n=1}{\sum }}x_{n}$	$\mathrm{Median}\;\frac{N+1}{2}$
Standard Deviation $\left(\sigma_{x}\right)=\frac{1}{N-1}\underset{n=1}{\sum }{\left(x_{n\;}-\mu_{x}\right)}^{2}$	Root Mean Square $x_{rms}=\sqrt{\frac{1}{n}\left(x_{1}^{2\;}+x_{2}^{2\;}+\ldots +x_{n}^{2\;}\right)}$
$\mathrm{Skewness}\;\frac{\sum_{n=1}^{N}{\left(x_{n}-\mu_{x}\right)}^{3}}{\left(N-1\right)\sigma_{x}^{3}}$	$\mathrm{Kurtosis}\;\frac{\sum_{n=1}^{N}{\left(x_{n}-\mu_{x}\right)}^{4}}{\left(N-1\right)\sigma_{x}^{4}}-3$
$\mathrm{Energy}\;\overset{N}{\underset{n=1}{\sum }}x_{n}^{2}$	$\mathrm{Sample}\;\mathrm{Entropy}\;SampEn\left(m,r,N\right)=-\ln\left[\frac{B^{m+1}\left(r\right)}{B^{m}\left(r\right)}\right]$
$\mathrm{Variance}\;\;\;Var\left(X\right)=E\left[{\left(X-\mu \right)}^{2}\right]$	$\mathrm{Maximum}\;\max\left(x_{n}\right)$
$\mathrm{Minimum}\;\min\left(x_{n}\right)$	$\mathrm{Hurst}\;E\left[\frac{R\left(n\right)}{S\left(n\right)}\right]=C_{n}^{H}\;as\;n\to \infty$
$\mathrm{Power}\;x\left(t\right)=\frac{1}{N}\overset{N}{\underset{i=1}{\sum }}p_{i}$	$\mathrm{Harmonic}\;\mathrm{mean}\;m=\frac{n}{\sum_{i=1}^{n}\frac{1}{x_{i}}}$

**Table 2:** EOG feature selection with ANOVA.

Features	Right Eye (*p* < 0.05)	Left Eye (*p* < 0.05)
Mean	0.000	0.000
Median	0.868	0.721
Maximum	0.007	0.025
Minimum	0.013	0.041
Power	0.014	0.180
Energy	0.001	0.005
Hurst	0.000	0.000
Variance	0.019	0.065
RMS	0.182	0.337
SD	0.182	0.337
Sample entropy	0.000	0.000
Harmonic Mean	0.582	0.850
Trimmean	0.615	0.673
Skewness	0.210	0.052
Kurtosis	0.692	0.106
Mode	0.000	0.000

**Table 3:** Performance of EOG showed in hypovigilance state detection.

Hypovigilance Detection	EOG Left Eye			EOG Right Eye		
	SVM	KNN	Ensemble	SVM	KNN	Ensemble
ND	80.2%	84.4%	90.2%	80.2%	86.8%	94.6%
NV	82.2%	77.5%	94.6%	82.4%	80.2%	96.3%
NC	71.3%	73.5%	89.0%	75.7%	89.0%	98.7%
NF	84.6%	85.7%	89.0%	81.3%	85.7%	90.7%
NDF	73.5%	77.9%	91.0%	77.9%	79.0%	91.3%
NDV	75.7%	84.6%	88.0%	79.0%	83.5%	90.2%
NDC	76.8%	82.4%	87.9%	72.4%	83.5%	93.5%
NDVC	88.0%	66.8%	82.7%	68.0%	72.4%	91.3%
NDVF	66.8%	78.0%	90.2%	71.3%	73.5%	82.4%
NDCF	77.9%	81.3%	83.5%	74.6%	86.8%	85.7%
NDVCF	69.0%	76.8%	86.6%	71.3%	77.9%	90.9%

[N-Normal, D-Drowsy, V-Visual inattention, C-Cognitive inattention, F-Fatigue].

**Table 4:** Performance of EOG in hypovigilance state detection.

Performance of Hypovigilance Detection on the Fusion of Classes							
	Classifier	Normal	Drowsy	Fatigue	Visual Inattention	Cognitive Inattention	Average
Before PCA	SVM	69.5	77.5	71.3	81.6	79.2	75.9%
	KNN	69.5	89.5	81.3	93.6	95.2	85.4%
	Ensemble	83.5	91.5	82.5	92.6	95.3	89.3%
After PCA	SVM	71.5	77.5	73.3	83.6	80.2	76.8%
	KNN	83.5	91.3	82.3	92.4	95.1	89.1%
	Ensemble	85.5	92.3	83.3	93.4	96.1	90.9%

**Table 5:** Comparison of performance with the related works.

Reference	Measures	Techniques	Detection	Accuracy
[45]	Physiological (EOG)	Neural network-based sampling with a greater optimized cross-sampling approach	Fatigue and Drowsiness	Blink, blink duration, eyelid location, PERCLOS are detected with few percent error
[46]	Physiological (EEG, EOG)	Linear trend removal, power spectral density (PSD)	Hypovigilance	Mean test error 26–32% for subjective and objective labels
[47]	EEG, forehead EOG	Double layered neural network with subnetwork nodes (DNNSN)	vigilance	RMSE/COR 0.11/0.79, 0.12/0.74, 0.08/0.86
[48]	EOG	Fuzzy logic	Drowsiness	Drowsy state: mean-74.18, SD-59.53425, alert state: mean-57, SD-14.70654
Proposed method	Physiological (EOG)	Feature reduction/fusion techniques (PCA)	Normal, fatigue, visual and cognitive inattention, drowsiness	Hypovigilance detection–90.9% accuracy, 79.8% sensitivity, 93.5% specificity, 81.6% precision, 9.1% error rate

## Data Availability

The datasets used and/or analyzed during the current study are available from the corresponding author on reasonable request.
